# Fatigue in taxi drivers and its relationship with traffic accident history and experiences: a cross-sectional study in the north of Iran

**DOI:** 10.1186/s12889-024-18044-5

**Published:** 2024-02-20

**Authors:** Enayatollah Homaie Rad, Marjan Hosseinnia, Nima Mousavi, Arian Shekari, Leila Kouchakinejad-Eramsadati, Naema Khodadadi-Hassankiadeh

**Affiliations:** 1 Social Determinants of Health Research Center, Trauma Institute, Guilan University of Medical Sciences, Rasht, Iran; 2 School of Pharmacy, Department of clinical and administrative sciences, Notre Dame of Maryland University, Baltimore, MD USA; 3 School of Medicine, Guilan University of Medical Sciences, Rasht, Iran; 4 Guilan Road Trauma Research Center, Trauma Institute, Guilan University of Medical Sciences, Rasht, Iran

**Keywords:** Fatigue, QOL, Driver, Traffic accident, Injury

## Abstract

**Background:**

The monotonous nature of work, long driving duration, and working overload hours cause frequent fatigue in taxi drivers. A high prevalence of fatigue is associated with traffic accidents. However, the risk factors associated with taxi driver fatigue are unclear. Therefore, the present study aims to determine the rate of fatigue in taxi drivers and its relationship to their traffic accident experience.

**Methods:**

In this descriptive-analytical study, 400 taxi drivers in the city of Rasht were registered in Taxi association selected through random sampling and entered into the study based on inclusion criteria. Data was collected through a researcher-made questionnaire reliable and valid by two medical students. The statistical analysis used ordinal data and a Poisson regression model with SPSS software version 21, with a significance level set at 5%.

**Results:**

The driver fatigue self-reported was directly and significantly related to alcohol consumption (OR = 3.43, 95% CI 1.01–11.62) and had a significant and inverse relationship with smoking (OR = 0.50, 95% CI 0.32–0.76), being married (OR = 0.08, 95% CI 0.01–0.40) and driving experience there was (OR = 0.96, 95% CI 0.94–0.98). Drivers’ sense of quality of life (QOL) was directly and significantly related to smoking (IRR = 1.43, 95% CI 1.28–1.59), education level under diploma (IRR = 2.41, 95% CI 1.43–4.06) diploma (IRR = 2.06, 95% CI 1.21–3.48) and bachelor (IRR = 2.42, 95% CI 1.36–4.29) and there was a significant and inverse relationship with age (IRR = 0.98, 95%CI 0.98–0.99). There was a significant relationship between the number of traffic accidents in the past year with the level of bachelor’s degree (IRR = 3.10, 95% CI 1.43–6.76) and driving experience (IRR = 1.03, 95% CI 1.02–1.04 and inverse relationship between the number of traffic accidents in the past year and the QOL sense (IRR = 0.96, 95% CI 0.93–0.99) and the working hours (IRR = 0.96, 95% CI 0.94–0.99).

**Conclusion:**

Legislators and policymakers should pay more attention to fatigue in single and inexperienced taxi drivers. Regarding the QOL, pay attention to drivers with high education and older. To reduce the number of crashes, pay more attention to drivers with a bachelor’s degree and less driving experience and improve the feeling of QOL.

## Background

Traffic accidents with much life, financial, and psychological burden still occupy first place in the ranking of traumas in north Iran [1, 2]. People with driving jobs are vulnerable to traffic accidents [3]. In Iran, on average, 38.03% of taxi drivers reported being involved in at least one traffic accident during the last year [4].

Driver fatigue as a problem in the modern world has attracted increasing attention. This problem has been given special attention by transportation companies in order to protect the employees, passengers, and economic stability of these companies. According to an International Road Traffic Accident Data (IRTAD) report from 38 countries, 26 countries report a percentage of fatal crashes due to fatigue in their reports, pointing out that there are far more occupational accidents among drivers [5]. The mean fatigue score in Iran in the defined taxi drivers was 58.60% [6].

In addition, a comprehensive crash analysis estimates that 10 to 20% of serious crashes are related to fatigue [7]. However, official statistics underestimate this effect because drivers involved in traffic accidents may not be able to recognize their fatigue or may not be able to express it for any reason [7, 8].

Fatigue manifests itself during a task’s long-term performance and is defined as “unwillingness to continue to perform an ongoing task“ [7]. There are two types of fatigue: one due to long hours of driving, which is called time-to-task fatigue, and the second form of fatigue from other sources, including insomnia, circadian rhythm failure, and other pre-driving functions called carry-over fatigue [9].

Fatigue is associated with decreased physiological arousal, slowness of sensory-motor functions, and damage to the information processing process, making it difficult for drivers to respond in emergencies and unusual and unexpected situations [7]. Fatigue reduces attention and alert reaction time and is a significant safety hazard in the transportation industry [10].

Driving as a skill activity requires constant attention and quick reaction [7]. Driver fatigue is more prevalent in professional drivers such as taxi drivers. Because they often have long periods of driving under pressure, lack of sleep, and circadian rhythm failure [11]. Although corrective factors should be emphasized when making preventive interventions, little is known about how fatigue, sleep, and health status are associated with driving risk in people with driving jobs. It must be addressed first [3].

Li et al. (2019) discovered the most critical factors associated with fatigue-related accident risk (FRAR) for taxi drivers. They reported that the driving hours of high-risk taxi drivers were significantly longer on a working day. They had less rest, less driving experience, and more confidence that they were resistant to fatigue [12].

In Iran, the taxi association, under the supervision of the municipality, is in charge of taxi management. Most taxi drivers are men, and as a means of public transportation, taxis have become a main arm of transportation and play an essential role in people’s daily urban trips in urban areas of Iran. In Iran, taxi drivers do not need to go through training courses and special qualifications, which means that anyone with a regular driver’s license and no criminal record can work as a taxi driver. Since the taxi fares are of the government tariff class and relatively low, taxi drivers are often low-income groups who have to spend long hours in this monotonous and tedious job to meet their financial needs and long hours of daily life [4, 6]. Finding themselves in overload hours and traffic congestion, these and other unknown factors cause these drivers to experience more fatigue, have a lower quality of life, and commit more risky driving compared to the general population [3, 7].

Taxi drivers worldwide often have very long driving hours and experience constant fatigue. Severe fatigue affects the safety motivation of taxi drivers. Driver fatigue reduces his motivation to adhere to driving safety principles [13]. In an Iranian study, job satisfaction scores had a positive association with rest/exercise hours and the income of taxi drivers. Moreover, there is a negative relationship between driving hours and education level [6]. The results from a study on Iranian taxi driver behavior showed that having a high annual driving mileage and a high number of daily taxi work hours were positively associated with traffic accidents [4]. However, the key factors differentiating taxi drivers from high/low traffic accident risk are unknown in northern Iran. Therefore, the present study aims to determine fatigue, QOL sense, traffic accident history, and risk factors for each taxi driver in north Iran.

## Methods

The present study was a descriptive-analytical cross-sectional study conducted in 2021 in Guilan province. After obtaining the code of ethics (IR.GUMS.REC.1399.572) from the ethics committee and the approval of the vice chancellor for research of Guilan University of Medical Sciences, the researchers, who had been two trained final year medical students, were introduced to Taxi Association by the Road Trauma Research Center. Considering the inclusion criteria, they collected data. All the taxi drivers were male, and other criteria included employment as a taxi driver for at least 12 months. (So, the answer to the traffic accident history in the past year is related to the time of employment as a taxi driver. (having a literate minimum reading and writing to respond to the content of the questionnaires and ethical considerations to Persian language and regional language. All taxi drivers participated in the study by their agreement, and they even wanted to give their mobile numbers. Any driver who reportedly is currently being treated for sleep disorders or used any sedatives or tranquilizers was excluded from the study (Because the report of variables of fatigue, sleep, and QOL or even traffic accidents may be affected by the use of these drugs.) [14, 15].

### Sample

Six thousand taxi drivers working on 82 routes in the city of “Rasht” were registered with the Taxi Association. Among these registered numbers, 400 samples were randomly selected using random software. Excel’s randomization command was used to determine the random case lists (RANDBETWEEN).

After the taxi drivers’ association coordinated with each of these drivers, the researchers introduced themselves to them and, after stating the objectives of the study, assured the participants that their personal information was considered confidential and anonymously entered into the analysis (however, most of them entered their names on the questionnaire). They were also assured that they could cancel completing the questionnaire anytime. After the taxi driver verbally stated that he was satisfied with participating in the study (the response rate was %100), they handed over the questionnaire to the driver. They stayed with him to answer vague questions and issues. The sampling process took approximately three months.

### Measures

#### Demographic-contextual characteristic

These include age, sex, body mass index (BMI), underlying diseases officially diagnosed by doctors (diabetes, hypertension, heart disease, cerebrovascular disease, gastrointestinal diseases, lung disease, musculoskeletal disorders, nervous system disorders, cancer, and other cases), smoking status, alcohol consumption, and exercise. The researcher calculated BMI using the height and weight reported by the driver. In the case of regular exercise, if regular exercise was done three or more times a week, “yes,” and otherwise, “no.”

#### Job characteristics

The amount of experience was based on the year and month who worked as a driver and the hours worked per day from the start to the end on most days.

#### Driver fatigue

Questions: 1- The time of onset of fatigue while driving (fatigue has started a few hours after the start of driving), 2- They rest after several consecutive hours of driving, 3- The duration of this rest (mean rest time), 4- The amount of fatigue (with a 5-point Likert scale, with one representing never and five representing always).

#### Details of the crash

Participants also reported any details of the traffic accident in the past 12 months (including the number of traffic accidents, whether the cause of the traffic accident was fatigue, and the uninterrupted driving hours before the crash).

#### QOL

The following single question was measured on a visual analog scale (VAS) (“How do you rate your QOL sense over the past week?” Answers with a score of 8 and above indicated an excellent sense of QOL, and answers of 7 and below indicated a low QOL sense [16].

#### Attitude to fatigue

Evaluated by three questions: (1) the effect of fatigue on driving performance (with a 5-point Likert scale, with 1 indicating no ill effects and five indicating a very undesirable), (2) How fatigue affects their driving performance (less overtaking, keeping long distance, lack of traffic awareness, reduced speed, speed up, lack of lateral control, slow reaction, and others.) and possible reasons that make you tired while driving: (Night driving, heavy food, noise in taxis, adverse taxi temperature, heavy traffic, driving on even track, short sleep time, long driving time, lack of sleep quality, other.)

#### Methods to deal with fatigue

Adaptation techniques were categorized using the article by Meng et al. (2015), and an “other” option was added because taxi drivers may use fatigue-specific coping techniques in taxi drivers. Take a short nap, stop the vehicle and exercise, eat some snacks, drink coffee/tea or energy drinks, drink water, smoke, open car windows, listen to the radio, listen to music, talk on the cell phone, talk to the passenger, washing the face, changing the position of the chair, watching the scenery, thinking, singing a song and more [7].

To validate this researcher-made checklist, it was first examined by five academic staff members of the Road Trauma Research Center and then revised and modified by five other experts. The reliability of the checklist was checked with 30 subjects and confirmed by the research team. The checklist has had a good test and retest (*r* = 0.78).

### Data analysis

Descriptive variables were reported as frequency (percentage) or mean (standard deviation). To confirm the final model of each fatigue risk index, we used ordinal regression in which the amount of fatigue was the dependent variable. The analysis was performed with SPSS version 21, and the significance level was set at the significance level of *P* < 0.05.

## Results

The mean age of 400 taxi drivers was 51.40 ± 10.43 years, the minimum age was 26, and the maximum age was 75 years. The majority were married, 387 (%96.8), and the mean height and weight were 172.55 ± 8.68 cm and 80.75 ± 14.68 kg, respectively. 110 (%27.5) drivers were smokers, and 11 (%2.8) were alcohol users. 304 (%76) drivers had no history of underlying disease (Table 1).

**Table 1:** Descriptive characteristics of taxi drivers (n = 400)

Variable	M ± SE
**Age (year)**	51.40 ± 10.43
**Height (cm)**	172.55 ± 8.68
**Weight (kg)**	80.75 ± 146
**Marital status**	N (%)
Single	9(2.25)
Married	387(96.75)
Widow	4(1)
**Education level**	
Illiterate	9(2.25)
Under Diploma	235(58.75)
Diploma	140(35)
Bachelor	16(4)
**Smock use**	
Yes	(027.5)110
No	290(72.50)
**Alcohol consumption**	
Yes	11(2.75)
No	389(97.25)
**Underlying Disease**	
Cardiovascular	21(5.25)
High blood pressure	30(7.50)
Kidney and urinary disease	14(3.50)
Digestive	9(2.25)
Diabetes	15(3.75)
Nervous disorders	7(1.75)
None	304(76)

Moderate fatigue was the most common type of reported fatigue, which was identified in the majority of married drivers160 (41.3), smokers 56 (50.9), and consumption of alcohol 4 (36.4) (Table 2).

Pearson Chi-Square showed that there is a statistically significant relationship between smoking (p-value 0.003), marital status (p-value 0. 000), and driving experience (p-value 0.000) with the QOL of these drivers. There was no statistically significant relationship between alcohol consumption (p-value 0.350) and quality of life.

**Table 2:** Demographic variable of fatigue the taxi drivers (n = 400)

Fatigue rate					
Variable	Never	Low	Moderate	Much	Very much
**Marital status**					
Single	0(0)	0(0)	3(33.3)	0(0)	6(66.7)
Married	20(5.2)	51(13.2)	160(41.3)	96(24.8)	60(15.5)
Widow	0(0)	0(0)	4(100)	0(0)	0(0)
**Smoke use**					
Yes	3(2.7)	20(18.2)	56(50.9)	21(19.1)	10(9.1)
No	17(5.9)	31(10.7)	107(36.9)	79(27.2)	56(19.3)
**Alcohol consumption**					
Yes	0(0)	0(0)	4(36.4)	4(36.4)	3(27.3)
No	20(5.1)	51(13.1)	159(40.9)	96(24.7)	63(16.2)

### Information about daily work

The mean driving experience in the subjects was 16.80 ± 9.73 years. They worked an average of 12.26 ± 2.20 h a day, and the average start and end of work in people was about 7:36 and 18:51. Most drivers (%53) drove more than 12,000 km a year. The mean rest time during work was 75.75 ± 42.39 min. Two hundred fifty drivers (%62.5) did regular exercise while working.

### Fatigue information

The most incredible feeling of fatigue while working was at 15:51.163(%40.8) reported moderate fatigue, and the highest effect of fatigue on driving performance was moderate 169 (42.3%). The mean QOL sense was 3.75 ± 2.23. The effect of fatigue on driving performance was reported in 109 (%27.3) speed reduction, and the most common cause of driver fatigue was heavy traffic 192 (%48). Most drivers believe drinking tea or coffee reduces fatigue 228 (%57). Most crashes are just minor car damage 201 (%50.3). The majority of people, 180 (%45), did not consider fatigue to cause their crash (Table 3).

**Table 3:** Data on the social/ work status of all the taxi drivers (n = 400)

Variable	M ± SE
**QOL sense**	3.75 ± 2.23
**Driving hours before the traffic accident**	11.33 ± 6.67
**Fatigue rate**	**N (%)**
Never	20(5)
Low	51(12.75)
Moderate	163(40.75)
Much	100(25)
Very much	66(16.50)
**The effect of fatigue on driving performance**	
None	20(5)
Low	87(21.75)
Moderate	169(42.25)
Undesirable	53(13.25)
Very undesirable	71(17.75)
**The type of fatigue affects performance**	
Less overtaking	47(11.75)
Keep a long distance	16(4)
Lack of traffic awareness	12(3)
Reduce speed	109(27.25)
Speed up	8(2)
Lack of lateral control	7(1.75)
Slow React	63(15.75)
Other	138(34.50)
**Causes of driver fatigue**	
Night driving	16 (4)
Heavy food	0(0)
Noise in taxis	9 (2.25)
Adverse taxi temperature	0(0)
Heavy traffic	192 (48)
Driving on an even track	14 (3.50)
Short sleep time	28 (7)
Long driving time	47 (11.75)
Lack of sleep quality	15 (3.75)
Other	79(19.75)
**Dealing with fatigue methods**	
Short nap	97 (24.25)
Stop and exercise	56 (14)
Drink coffee/tea or energy drinks	228 (57)
Other	19 (4.75)
**Traffic accident history in the past year**	
Number of damage	201 (50.25)
Number of injuries	12 (3)
Number of occupant mortality	42 (10.50)
Without traffic accident	145 (36.25)
**Fatigue is the cause of this traffic accident**	
Yes	143 (35.75)
No	257 (64.25)

QOL: Quality of life

In the context of ordinal logistic regression, driver fatigue exhibits a clear and statistically significant positive correlation with alcohol consumption (OR = 3.43, 95% CI 1.01–11.62). Conversely, a significant and negative correlation is evident in the relationship between driver fatigue and smoking (OR = 0.50, 95% CI 0.32–0.76). Furthermore, a noteworthy and inversely significant connection exists between driver fatigue and being married (OR = 0.08, 95% CI 0.01–0.40). The association between driver fatigue and driving experience is similarly marked by a significant inverse relationship (OR = 0.96, 95% CI 0.94–0.98) (Table 4).

**Table 4:** Relationship between fatigue with BMI, age, smoke use, exercise rate, driving experience, alcohol consumption, marital status and education level (n = 400)

Variables		Odds Ratio	Z	P-Value	95%CI	
					low	up
BMI		7.1	1.83	0.068	2.01	0
Age		1.01	1.32	0.186	0.99	1.03
Smock use		0.50	-3.23	0.001*	0.32	0.76
Exercise rate		1.30	1.27	0.204	0.86	1.97
Driving experience		0.96	-3.23	0.001*	0.94	0.98
Alcohol consumption		3.43	1.98	0.048*	1.01	11.62
Marital status	Married	0.08	-3.12	0.002*	0.01	0.40
	Single	0.22	-1.36	0.174	0.02	1.91
Education level	< Diploma	0.75	-0.43	0.665	0.21	2.66
	Diploma	1.23	0.32	0.751	0.34	4.41
	Bachelor	0.70	-0.73	0.670	0.14	3.42

Alternatively, Odds Ratio, p- Value = probability value, *statistically significant at the 0.05 level (2-tailed), CI: confidence intervals, adjusted for BMI, age, smock use, exercise rate, driving experience, alcohol consumption, marital status, and education level

Poisson regression estimator showed that drivers’ QOL sense was directly and significantly related to smoking (IRR = 1.43, 95% CI 1.28–1.59) and education levels under diploma (IRR = 2.41, 95% CI 1.43–4.06) diploma (IRR = 2.06, 95% CI 1.21–3.48) and bachelor (IRR = 2.42, 95% CI 1.36–4.29). There was a significant and inverse relationship between QOL sense and age (IRR = 0.98, 95% CI 0.98–0.99) (Table 5).

**Table 5:** Relationship between QOL sense with BMI, age, smoke use, exercise rate, driving experience, alcohol consumption, marital status, and education level (*n* = 400)

Variables		IRR	Z	P-Value	95%CI	
					low	up
BMI		6.66	1.90	0.058	0.03	1.30
Age		0.98	-5.52	0.0001*	0.98	0.99
Smoke use		1.43	6.53	0.0001*	1.28	1.59
Exercise rate		0.94	-0.96	0.337	0.85	1.05
Driving experience		0.99	-1.00	0.315	0.99	1.00
Alcohol consumption		0.90	-0.67	0.504	0.66	1.22
Marital Status	Married	1.04	0.27	0.786	0.75	1.45
	Single	0.72	-0.96	0.337	0.37	1.39
Education level	> Diploma	2.41	3.33	0.001*	1.43	4.06
	Diploma	2.06	2.70	0.007*	1.21	3.48
	Bachelor	2.42	3.03	0.002*	1.36	4.29

IRR: Incidence Rate Ratio, p- Value = probability value, *statistically significant at the 0.05 level (2-tailed), CI: Confidence Intervals, adjusted for BMI, age, smock use, exercise rate, driving experience, alcohol consumption, marital status, and education level

Poisson regression estimator showed that there was a direct and significant relationship between the number of traffic accidents in the past year and the education level of bachelor’s degree (IRR = 3.10, 95% CI 1.43–6.76) and driving experience (IRR = 1.03, 95% CI 1.02–1.04). There was a significant and inverse relationship between the number of traffic accidents in the past year and the QOL sense (IRR = 0.96, 95% CI 0.93–0.99) and the working hours (IRR = 0.96, 95% CI 0.94–0.99) (Table 6).

**Table 6:** Relationship between the number of traffic accidents with BMI, age, fatigue, QOL sense, working hours, driving experience and education level (n = 400)

Variables		IRR	Z	P-Value	95%CI	
					low	up
BMI		4.36	-0.25	0.805	1.9	9.95
Age		0.99	-1.45	0.147	0.98	1.00
Fatigue		1.06	1.34	0.179	0.97	1.17
QOL sense		0.96	-2.04	0.041*	0.93	0.99
Working hours		0.96	-2.41	0.016*	0.94	0.99
Driving experience		1.03	5.53	0.0001*	1.02	1.04
Education level	>Diploma	1.49	1.15	0.248	0.75	2.94
	Diploma	1.50	1.18	0.238	0.76	2.98
	Bachelor	3.10	2.86	0.004*	1.43	6.76

IRR: Incidence Rate Ratio, p- Value = probability value, *statistically significant at the 0.05 level (2-tailed), CI: Confidence Intervals, adjusted for BMI, age, fatigue, QOL sense, working hours, driving experience, and education level

## Discussion

In the present study, driver fatigue was directly and significantly related to alcohol consumption. That is, taxi drivers who stated that they had a history of alcohol consumption saw a history of alcohol consumption as a factor in increasing fatigue. Considering that only 11 drivers (%2.8) reported having a history of alcohol consumption. The low rate of their reporting is likely due to the Islamic laws of the country, which make it illegal to consume alcohol in any amount and under any title. In the study of Kwon et al. (2019), alcohol consumption is associated with speed and traffic accidents [3]. A previous study reported that taxi drivers had risky driving behaviors while fatigued and driving. At the same time, alcohol is drunk, increases the probability of accidents with injuries by 16.7% and 7.9%, respectively. Because alcohol increases their resistance to fatigue, they continue to drive dangerously for many hours [17].. No study was found on Iranian taxi drivers on alcohol consumption and its association with fatigue. Maybe it is because taxi drivers get fired if they drink alcohol or do not get a work permit at all, and these laws are even more tightly regulated and enforced in other countries. The results of an experimental test also showed that different levels of alcohol and fatigue impose different effects on driver’s safety performance [18]. In the present study, because the self-report of alcohol consumption was low, we could not be very sure about the relationship obtained.

In addition, there was a significant and inverse relationship between the rate of expression of taxi drivers’ fatigue and smoking. That is, our drivers stated that smoking has a positive effect on reducing their fatigue. Similarly, in a survey study, the Chinese reported that taxi drivers smoke a lot to relieve fatigue for reasons such as long working hours, high intensity of work, and the need to renew energy. Moreover, in a study in Singapore, the proportion of taxi drivers with risk factors such as smoking and a sedentary lifestyle was higher than the general population [19]. In the study, Kwon, Kim. et al. (2019), smoking was associated with speeding and traffic accidents [3]. In a study of taxi drivers, the acute activation of the sympathetic response through smoking, especially at night, can have a powerful effect on the progression of coronary atherosclerosis or may even lead to cardiovascular events in less experienced and younger taxi drivers who are healthy [20]. However, the mental need of these drivers for nicotine in cigarettes can have contributed to their positive statements about the excellent relationship between smoking and fatigue.

This study showed a significant and inverse relationship between the fatigue of taxi drivers and being married. An Iranian study reported that factors such as the marital status of taxi drivers can affect taxi traffic accidents [4]. The presence of more needs, such as more financial needs of married taxi drivers, has caused them to continue driving for long hours and suppress the feeling of fatigue. Their self-declaration of fatigue is, therefore, low.

On the other hand, in the present study, there was a significant and inverse relationship between taxi drivers’ fatigue level and driving experience. In a similar study, taxi drivers at risk of fatigue had significantly more driving hours per day, lower rest ratio, less driving experience, and greater confidence in their resistance to fatigue [12].In another study, older taxi drivers performed more dangerous behaviors and experienced more traffic accidents [21].

In the present study, the better perceived QOL sense of drivers was directly and significantly related to smoking. No study has been found that explicitly addresses QOL sense smoking among taxi drivers. Similarly, a meta-analysis found no significant differences in all-cause mortality and health risk among people who reduced smoking [22]. Moreover, another study proves that former smokers are statistically indistinguishable from never-smokers regarding reduced work productivity [23]. In a study, smoking taxi drivers’ QOL scores decreased in physical health but increased in the environment [24]. Paradoxically, a study reported a relationship between drivers’ QOL and pain and discomfort of dependence on medication or therapy methods and ability to work [25]. Therefore, while we expected smoking to hurt the QOL of our drivers, the statements of the drivers themselves want to state that smoking not only does not harm their QOL but also makes it better. Monotony, uncertain working conditions, and lack of professional value sometimes affect a person’s mental performance. These factors can negatively affect the evaluation score in the psychological and environmental domains and the overall QOL score [26–28].

In addition, in the present study, the QOL sense of drivers was directly and significantly related to all levels of education. An Iranian study showed that the job satisfaction scores of taxi drivers had a negative relationship with their level of education [6]. In another study, in the study of the relationship between the education level variable and drivers’ quality of life, the p-value for the education level variable was close to the significance threshold, which was due to a weak relationship. Drivers with lower education levels reported more work-related accidents and lower quality of life [24]. Although in previous studies, drivers’ QOL was not significantly related to their level of education [28, 29], the previous studies explicitly clarified the importance of education. They thus reported that the solution to the problems of reckless and aggressive behavior of taxi drivers is to teach them to pay attention to traffic laws and ways to deal with fatigue and reduce risky behaviors [3, 7, 30].

In the present study, there was a significant and inverse relationship between QOL sense and the age of taxi drivers. This relationship is reversed in taxi drivers, and their QOL sense decreases with age. Similarly, in a study, motorcycle taxi drivers aged 40 and older reported 31% lower work capacity than younger drivers. However, the absenteeism rate was higher among motorcycle taxi drivers aged 21–29 and 30–39 years [31]. In another study, in the analysis of the relationship between demographic factors and quality of life, a statistically significant relationship was observed between age and quality of life, which means that older drivers had more work-related accidents and, as a result, reported a lower QOL [24].

This study showed that the number of traffic accidents in the past year was directly and significantly related to the level of bachelor’s degree. In an Iranian study, the higher educational background of a taxi driver hurts his aggressive violations [4]. In another study, high-risk and unsafe behaviors of taxi drivers were significantly associated with their level of education [32]. However, in a study of taxi drivers, education was not associated with injuries [33]. In northern Iran, those with a bachelor’s degree expect to have a higher job rank. It is possible that the mental distress due to job dissatisfaction of these people from their type of job subconsciously caused high-risk behavior while driving and increased their injury rate.

Based on the results, the number of traffic accidents in the past year was directly and significantly related to driving experience. However, in a study on Iranian taxi drivers, it was found that there is a significant relationship between driving experience and anger management. Also, there was a close correlation between the temperaments of taxi drivers’ driving offenses [34]. Previous studies have shown a significant relationship between driving experience and work experience with high-risk behaviors of taxi drivers [35, 36]. When the taxi driver is more experienced, he should ignore his fatigue while driving. He may even tend to resist fatigue more, continue high-risk driving for longer hours, and take less rest than other drivers.

This study revealed a significant and inverse relationship between the number of traffic accidents in the past year and working hours. Nonaligned, in one study, taxi drivers who worked more than 50 h per week engaged in more risky driving behaviors [37]. Also, in an Iranian study, high annual driving mileage and many daily taxi trips positively correlate with accidents [4]. What should be considered is not the number of working hours, but should be paid attention to working in overloaded hours and dangerous working hours such as at night.

In this study, the average rest time during work was 75 minutes. Our drivers felt the most tired while working at 15:51. This is when most employees are naturally taking a nap, and it is natural that taxi drivers are more tired and need rest. One study analyzed a set of factors related to taxi drivers' fatigue and taxi drivers’ traffic accidents over the past two years. They found a significant negative correlation between total mean rest time and traffic accident rate [38]. Phatrabuddha et al. (2018) reported that drivers who sleep less than 7 h and have poor sleep quality are more tired than those who sleep well enough. Drivers with higher drowsiness scores had more objective fatigue than those with lower drowsiness scores [39].

### Limitations

The limitation of the current study was that it needed a comparison group to compare the variables of taxi drivers with other professional drivers. In the north of Iran, in the inner city system, there are private taxis, such as telephone taxis and internet taxis (Snapp / Topsy), which have yet to be studied and compared. In addition, in most other studies, taxi drivers worked the night shift, so it was impossible to compare our study’s results with those of other countries. In the end, we could not enter all the potential confounding variables.

## Conclusion

This study reveals that the factors that tire our taxi drivers are not the opposite of long working hours but alcohol consumption, living alone, and long experiences working in this job. Being a taxi driver with a high education could even make a person feel right about the quality of life, so it is better to choose the level of education appropriate to this job rather than higher. In addition, elderly taxi drivers should be given more attention and care regarding why they feel a low quality of life. If they felt better about their quality of life, they would work longer hours, be more productive, and, more importantly, experience fewer traffic accidents. These results make it clear that the legislators and policymakers should establish laws and policies to correct the existing situation by knowing precisely the characteristics of these city taxi drivers who have been engaged in monotonous and tedious work for years with government prices. In all training programs under the title of preventing taxi drivers’ fatigue, it is necessary to teach valuable ways to deal with it and prohibit alcohol consumption as a method to relieve fatigue based on evidence. Choose marriage as a necessity of a healthy lifestyle in terms of psychological dimensions and suggest job diversification after a few years of work experience as an excellent way to relieve fatigue. In terms of creating a better feeling of quality of life, pay attention to older age groups. Provide training programs for taxi drivers with a bachelor’s degree or higher and experience in driving with low working hours and a lower quality of life. Further research in this field can investigate the effect of various intervention methods to relieve fatigue, improve the quality of life, and reduce taxi drivers’ accidents in older, single, bachelor, experienced taxi driver groups.

## Data Availability

All data generated or analyzed during this study are included in the submission.
